# Small Molecule-Induced Domain Swapping as a Mechanism for Controlling Protein Function and Assembly

**DOI:** 10.1038/srep44388

**Published:** 2017-03-13

**Authors:** Joshua M. Karchin, Jeung-Hoi Ha, Kevin E. Namitz, Michael S. Cosgrove, Stewart N. Loh

**Affiliations:** 1 Department of Biochemistry and Molecular Biology, State University of New York Upstate Medical University, 750 East Adams Street, Syracuse, NY, 13210, USA.

## Abstract

Domain swapping is the process by which identical proteins exchange reciprocal segments to generate dimers. Here we introduce induced domain swapping (INDOS) as a mechanism for regulating protein function. INDOS employs a modular design consisting of the fusion of two proteins: a recognition protein that binds a triggering molecule, and a target protein that undergoes a domain swap in response to binding of the triggering ligand. The recognition protein (FK506 binding protein) is inserted into functionally-inactivated point mutants of two target proteins (staphylococcal nuclease and ribose binding protein). Binding of FK506 to the FKBP domain causes the target domain to first unfold, then refold via domain swap. The inactivating mutations become ‘swapped out’ in the dimer, increasing nuclease and ribose binding activities by 100-fold and 15-fold, respectively, restoring them to near wild-type values. INDOS is intended to convert an arbitrary protein into a functional switch, and is the first example of rational design in which a small molecule is used to trigger protein domain swapping and subsequent activation of biological function.

A domain swap is defined as the replacement of a segment of a monomeric protein by the same segment from another copy of the same protein^1^,^2^,^3^. Nature uses domain swapping to create specific binding interfaces as well as encode for new or altered functions that appear only in the swapped state^4^,^5^,^6^,^7^,^8^. Swapping can result in closed, topologically circular complexes (frequently dimers) or, when uncontrolled, in open-ended polymeric structures that contribute to pathogenic conditions^9^,^10^,^11^,^12^.

As a protein engineering tool, domain swapping is relatively new and its capabilities are only beginning to be realized. It is inherently well-suited to generating self-assembling nanostructures and naturally monomeric proteins have been coaxed to swap in order to create functional biomaterials^13^,^14^. More recently, we and others have used engineered swapping to effect changes in biological activity of proteins, including ligand binding properties^15^,^16^,^17^ and intein-mediated protein splicing^18^.

What is largely missing from current designs is the ability to trigger domain swapping on-demand. This property will unlock the potential of domain swapping as a mechanism for protein conformational switching. The main obstacles to this goal are that the thermodynamic factors—and to a greater extent, the kinetic considerations—that drive proteins to swap are incompletely understood. How can one induce a protein to swap when the structures of the monomeric and swapped conformations and the interactions that stabilize them are nearly identical ? Historically, when a mutation or solution condition was found to result in swapping, that result was often unexpected and the underlying reasons were not clear. Elevated protein concentrations clearly favor a domain swap, but it is often impractical to raise bulk protein concentration above the already high local concentration of polypeptide segments present during intramolecular folding. Moreover, even if swapping can be made thermodynamically favorable, the protein may be kinetically trapped as a monomer because an unfolding reaction is required to swap. At minimum, a segment must detach from one monomer in order to insert into a second monomer, and at maximum the protein may need to globally unfold. The kinetic barriers to unfolding can be high and increasing protein concentration does not lower them. INDOS lowers these barriers by coupling unfolding to a ligand-induced folding reaction, as discussed below. In the present examples, we show that swapping occurs within minutes to hours after addition of triggering ligand, at protein concentrations in the low micromolar range.

Current efforts to rationally design swapping focus heavily on the hinge region—the segment that forms the crossover point between protomers—because it is the only region of the protein that is consistently and substantially different in the monomeric vs. swapped states^1^,^19^. Our strategy and that of some others is to engineer a hinge that stresses the conformation of the protein when it is monomeric but not when it is swapped. Linking that stress to a signaling event can then provide the triggering mechanism, as demonstrated by Woolley and co-workers in their design of a light-induced domain swapping switch^20^.

Our approach entails inserting a ‘lever’ protein into a surface loop or turn of a target protein (Fig. 1A). If the N-to-C terminal distance of the lever exceeds that of the C_α_–C_α_ distance between terminal residues of the loop, folding of the lever becomes structurally and thermodynamically coupled to unfolding of the target and vice versa^21^. Once the lever unfolds the target, the latter is free to refold in *trans* via domain swap^22^. In the resulting dimer or oligomer, all target and lever domains are folded and conformational strain is relieved. In doing so the lever protein becomes the hinge region of the dimer and is thus placed outside of the structure of the target protein. We further showed that this design can be used to turn on and off activity of the target domain^17^. Two separate lever-target fusion proteins were made wherein the function of the target domain (ribose binding protein, or RBP) was knocked out by point mutations N-terminal and C-terminal to the lever (ubiquitin) insertion site. Domain swapping resulted in dimers composed of one inactive RBP domain that contained both mutations, and one active RBP domain that contained none.

The induced domain swapping (INDOS) technology that we introduce here improves upon our earlier mechanism by making it responsive to binding of a ligand (the drugs FK506 or rapamycin). We establish the modularity of INDOS by demonstrating that the same ligand-binding lever domain (FK506 binding protein, or FKBP) can be used to regulate the biological activity of two target proteins: RBP and staphylococcal nuclease (SNase). We show that this on/off switch is efficient—FK506 activates SNase and RBP function by ~100-fold and >15-fold, respectively—and fully reversible.

## Results

### Model of triggerable domain swapping

**Figure 1:**
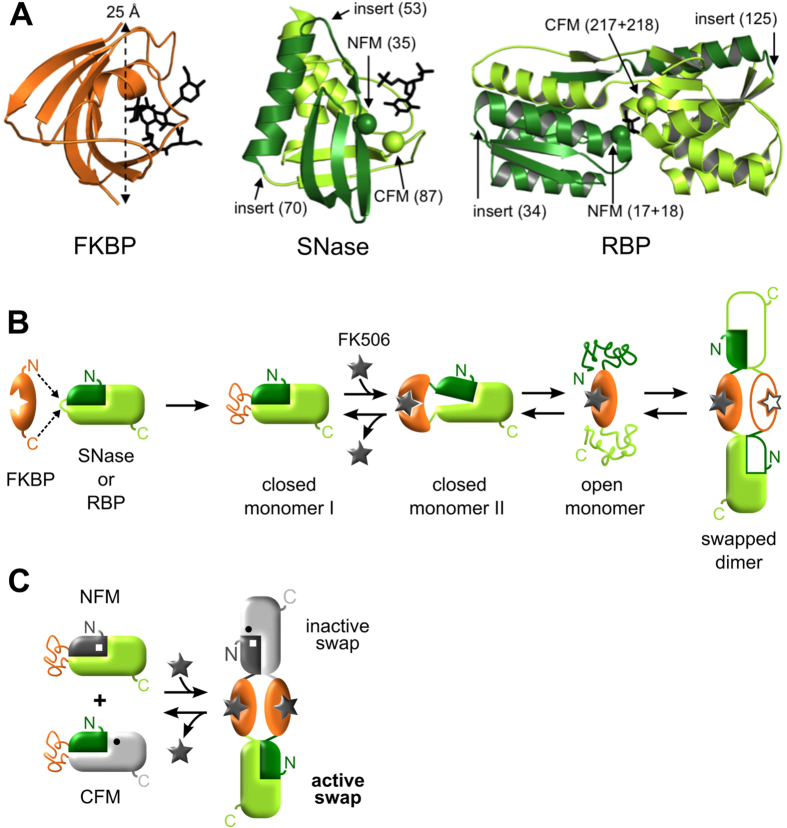
INDOS and domain-swapping bioswitches. Lever proteins are colored orange and target proteins are colored green. (**A**) Structures of the lever protein FKBP (PDB 1FKF) with its N-to-C distance indicated, and the target proteins SNase (PDB 3BDC) and RBP (PDB 2IOY) with their insertion positions and functional mutations noted. Bound ligands (FK506, the inhibitor pdTp, and ribose) are shown as black sticks in the FKBP, SNase, and RBP structures, respectively. (**B**) Proposed mechanism of INDOS. A triggerable lever (FKBP) is inserted into a surface loop of a target protein (SNase or RBP). The target domain is initially more stable than the lever domain and the fusion protein adopts the closed monomer I conformation in the absence of FK506. FK506 binding stabilizes the lever, resulting in the strained and transiently populated closed monomer II state. Conformational strain is relieved by unfolding to the open monomer state followed by refolding in *trans* to generate swapped dimers and oligomers. (**C**) INDOS is used to create bioswitches by introducing NFM (white square) and CFM (black circle) point mutations into the target protein, on either side of the lever, rendering the construct inactive (partially or completely gray structures). The scheme in panel B results in swapped dimers, which can consist of NFM-CFM heterodimers (shown) and NFM-NFM and CFM-CFM homodimers (not shown). Biological activity is restored in NFM-CFM heterodimers (green domain), whereas all monomeric species as well as NFM-NFM and CFM-CFM homodimers remain nonfunctional.

Our previous domain swap designs used ubiquitin as the lever protein. Because ubiquitin is very stable and does not bind a ligand with high affinity, it is a static lever that is always ‘on’. To make swapping controllable by a small molecule we employed a lever protein (FKBP) that binds the cell-permeant drug FK506 (K_d_ = 0.6 nM^23^) (Fig. 1A), and exploited the inherent coupling between binding and folding. The minimal sequence of events linking FK506 binding to domain swapping is illustrated in Fig. 1B. In the unliganded (apo) state, the fusion protein adopts the closed monomer I conformation in which the SNase and RBP target domains are folded and the FKBP lever domain is at least partially unfolded. Ligand binding stabilizes and folds FKBP, leading to the closed monomer II state. This structure is strained and is expected to be metastable. Strain is relieved when the target domain unfolds to yield the open monomer. Finally, open monomers refold via domain swap of the target domains to generate dimers and higher order oligomers.

We connected the above mechanism to regulation of protein function by creating two mutants of the target domain in which the function of each is knocked out by amino acid substitution. One mutation is placed N-terminal to the FKBP insertion point (N-functional mutant or NFM) in one copy of the fusion protein, and the other mutation is placed C-terminal to the FKBP insertion point (C-functional mutant or CFM) in the second copy of the fusion protein. When these constructs domain swap, one product contains one target domain in which the mutations have swapped out and biological activity has been restored (Fig. 1C). We inserted FKBP into positions 53 and 70 of SNase (denoted SF53 and SF70) and into positions 34 and 125 of RBP (designated RF34 and RF125) (Fig. 1A). For NFM/CFM we selected the R35A/R87A catalytic mutants of SNase^24^ and the (F17A + F18A)/(F217A + D218S) ribose binding mutants of RBP^25^,^26^,^27^ (Fig. 1A).

Of all protein species present in Fig. 1, only NFM-CFM hetero-swapped complexes possess biological function (Fig. 1C). All monomers are inactive by design, but NFM-NFM and CFM-CFM homo-swapped complexes also lack activity and constitute dead-end products. NFM-NFM and CFM-CFM complexes are virtually identical to the NFM-CFM structure depicted in Fig. 1C, and hence are not shown for the sake of clarity. It is reasonable to expect *a priori* that swapping will populate hetero- and homo-complexes according to their relative thermodynamic stabilities. Using this principle, it is possible to tune the populations to favor the NFM-CFM complex by the use of destabilizing mutations. If one pairs a second, destabilizing point mutation with each of the NFM and CFM mutations (N-terminal and C-terminal to the lever insertion site, respectively), then the only species in Fig. 1 that is not destabilized by this second set will be the active complex. The reason is that the destabilizing mutations will be swapped out along with the NFM and CFM mutations, leaving the resulting structure both active and stable (Fig. 1C). In the present case of the SF and RF constructs, the NFM and CFM mutations are themselves slightly destabilizing^17^,^28^, so we did not introduce any secondary mutations for the purpose of favoring the NFM-CFM complex.

### Structural and kinetic characterization of the swapping mechanism by circular dichroism

**Figure 2:**
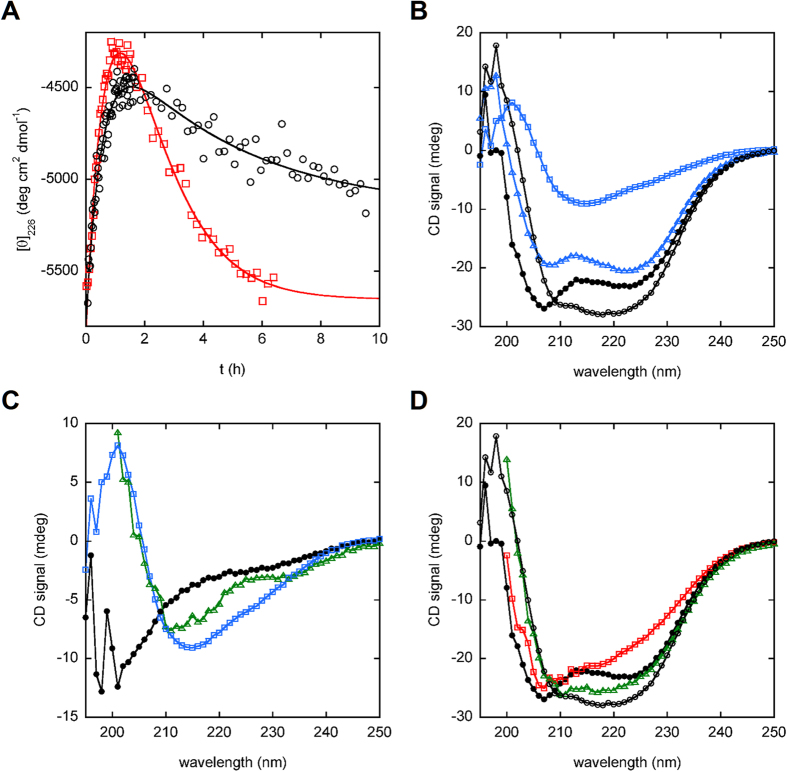
Kinetic and structural characterization of FK506-induced SF70 domain swapping. (**A**) After FK506 is added to apo SF70 at time zero, CD detects an initial unfolding event followed by a protein concentration-dependent refolding event. Black circles and red squares indicate protein concentrations of 2 μM and 30 μM, respectively. Solid lines are best fits of the data to a two-exponential function. (**B**) CD spectra of apo SF70 (closed black circles) and free SNase (blue triangles) are similar and indicate extensive α-helical content. The CD spectrum of free FKBP (blue squares) shows mostly β-sheet structure. The CD spectrum of apo SF70 is significantly different than the sum of free SNase and free FKBP spectra (open black circles), suggesting that the FKBP domain of apo SF70 is in a non-native conformation. (**C**) The CD difference spectrum of apo SF70 – free SNase (closed black circles) shows extensive random coil structure, whereas that of holo SF70 – free SNase (17 h incubation with FK506; green triangles) resembles the CD spectrum of free FKBP (blue squares). (**D**) After FK506 is added to apo SF70 (closed monomer I; closed black circles), the holo protein first unfolds to generate the open monomer (1.5 h; red squares) and then refolds to yield domain swapped dimers and oligomers (17 h; green triangles). Both SNase and FKBP domains appear to be natively folded in the domain-swapped species, as judged by the similarity of holo SF70 spectrum to the sum of free SNase and free FKBP spectra (open circles), and by the holo SF70 – free SNase difference spectrum (panel C).

To test the scheme in Fig. 1B we used circular dichroism (CD) to monitor kinetics of FK506-induced structural changes of SF70 at 2 μM and 30 μM enzyme concentration. The CD signal of SF70 at 226 nm originates predominantly from the α-helices of the SNase domain; the data do not report on conformational changes of the mostly β-sheet FKBP domain. Two kinetic phases are observed after adding FK506 (Fig. 2A). The first phase reflects an unfolding event with half-times (t_1/2_) of 40 min (2 μM protein) and 22 min (30 μM protein). We attribute this process to transition of closed monomer II to open monomer, in which the helical structure of the SNase domain is disrupted. Size exclusion chromatography (SEC) results (see following section) suggest that binding of FK506 and subsequent conversion of closed monomer I to closed monomer II occurs within the ~1 min dead time of the CD experiments. The second, rate-limiting phase reflects a folding step that likely corresponds to refolding of open monomers to domain-swapped complexes in which both FKBP and SNase domains are natively structured. The rate of this step is slower than that of folding of either free SNase or free FKBP and it increases with protein concentration (t_1/2_ = 3.1 h and 0.89 h at 2 μM and 30 μM, respectively). These findings indicate that the swapping rate is not limited by a unimolecular conformational change such as folding/unfolding. Instead, it appears to be at least partially dictated by the encounter of protein monomers. Since the observed rates are much slower than the diffusion-limited rate that would be estimated from the total protein concentration in the experiment (t_1/2_~1 s, assuming an association rate constant of 10^6^–10^7^ M^−1^ s^−1^), it is likely that the percentage of protein in the swapping-competent conformation is only a small fraction of the total protein concentration. The FK506-induced unfolding step in Fig. 2A may produce an ensemble of unfolded SNase conformations, only some of which are competent to swap.

To gain insight into the structure of apo SF70 we compared its CD spectrum with those of free SNase and free FKBP recorded at the same protein concentration (6.9 μM). Free SNase exhibits pronounced minima at 222 nm and 208 nm (Fig. 2B) arising from its largely α-helical structure (Fig. 1A), whereas free FKBP displays a weak minimum near 215 nm indicative of its predominantly β-sheet conformation. The SNase domain of apo SF70 appears to be well folded, as evidenced by the strong 222 nm and 208 nm minima. The spectrum of apo SF70 is, however, significantly different than the sum of the spectra of free SNase and free FKBP (Fig. 2B), suggesting that the FKBP domain of apo SF70 is in a non-native conformation. To characterize this conformation we subtracted the spectrum of free SNase from that of apo SF70 (Fig. 2C). The difference spectrum is characteristic of random coil, with the ellipticity becoming more negative below 210 nm rather than increasing in the case of α or β structure. These observations together suggest that the SNase domain of SF70 is folded while the FKBP domain is at least partially unfolded. This structure is consistent with closed monomer I (Fig. 1B).

To further test the mechanism in Fig. 1B, we added FK506 to the SF70 sample shown in Fig. 2B and recorded CD wavelength scans at times at which the open monomer and swapped dimer are populated (1.5 h and 17 h, respectively). The 1.5 h time point shows significant loss of helical signal compared to the apo protein, with the minimum at 222 nm becoming much less pronounced (Fig. 2D). By 17 h, not only has the helical signal at 222 nm been fully restored, the β-sheet region between 210–220 nm has become more intense than that observed in the apo sample, to the point where the holo SF70 spectrum is similar to that of the sum free SNase and free FKBP (Fig. 2D). When the free SNase spectrum is subtracted from that of the 17 h time point, the difference spectrum now more closely resembles that of free FKBP (Fig. 2C). All domains thus appear to be natively structured in the swapped species, as predicted by Fig. 1B.

### Determination of oligomeric population by analytical ultracentrifugation

**Figure 3:**
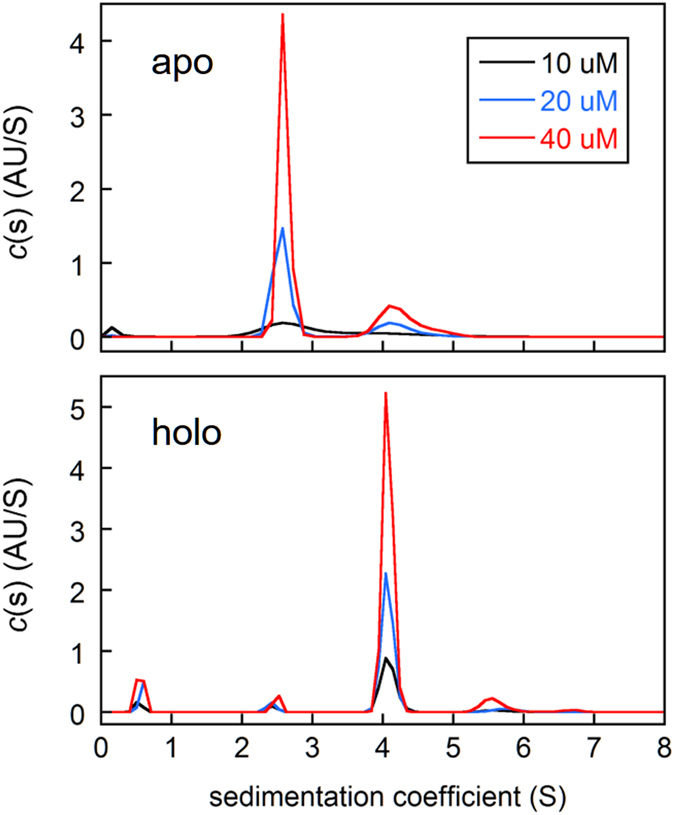
AUC reveals that SF70 is primarily monomeric in the absence of FK506 and dimeric in the presence of FK506. See Table S1 for sedimentation coefficients, MW_app_ values, and peak integrations.

We employed sedimentation velocity analytical ultracentrifugation (AUC) to further characterize the structures and oligomerization states of SF70 at 10 μM, 20 μM, and 40 μM concentration. At 20 μM and 40 μM, apo SF70 sediments as a major species of apparent molecular weight (MW_app_) 25.4 kDa (theoretical monomer MW = 29.943 kDa) and a minor species of MW_app_ = 53.5 kDa, indicating a mixture of 76–78% monomer and 22–24% dimer (Fig. 3; Table S1). Apo SF70 at 10 μM sediments as a single broad peak with a sedimentation coefficient (S) similar to that of the monomer peaks at 20 μM and 40 μM protein, but with a tail at higher S-values that precludes accurate estimation of MW_app_. After equilibration with FK506 for 24 h the monomer and dimer populations invert to 79–81% dimer and 2.5–6.1% monomer, with the fraction monomer decreasing with increasing protein concentration. Together with the CD data these results strongly support the model in Fig. 1B and reveal that swapping of holo SF70 produces mainly dimers at the enzyme concentrations tested.

Compared to SF70, SF53 exhibits an increased tendency to domain swap in the absence of FK506. 40 μM apo SF53 sediments as a mixture of monomer (51%), dimer (20%), and trimer or tetramer (12%) (Fig. S1; Table S1). As protein concentration is lowered to 20 μM the dimer and trimer/tetramer peaks become less well resolved, and at 10 μM they coalesce into a single broad peak with an S-value in between that of dimer and trimer/tetramer. Dimer and higher-order oligomers therefore appear to interconvert on the time scale of the AUC experiment. Addition of FK506 to SF53 causes oligomeric species to increase in intensity at the expense of the monomer peak. Unlike SF70, however, a significant population of monomer remains, suggesting that the drug does not trigger the swapping process as effectively for SF53.

### Regulation of SNase enzymatic activity by FK506

**Figure 4:**
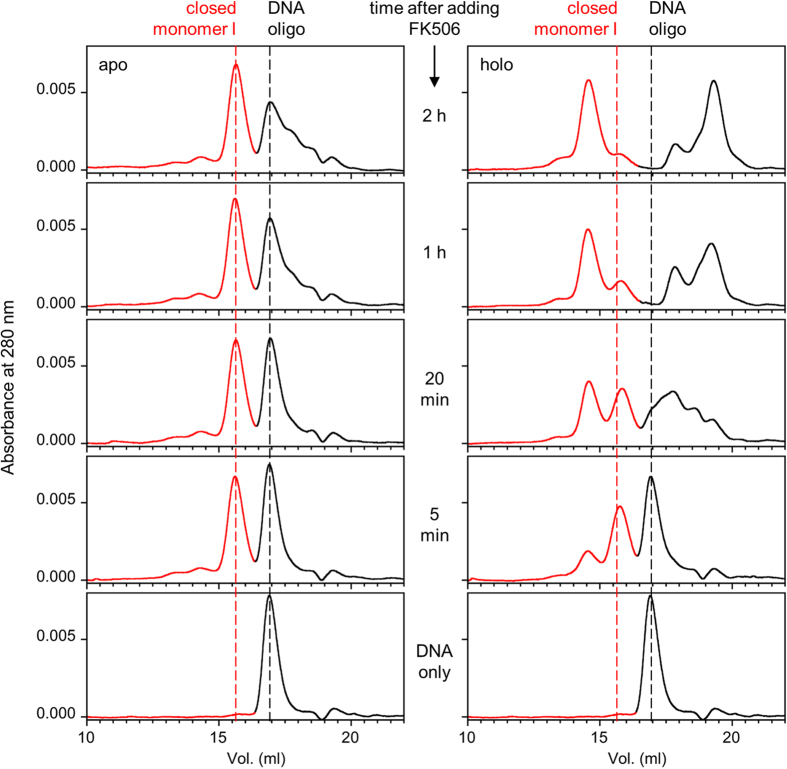
FK506 induces dimerization of SF70 and activates enzymatic activity. Following addition of FK506 or DMSO vehicle to a mixture of SF70-NFM + SF70-CFM, size exclusion chromatograms show conversion of protein monomers to domain-swapped dimers (red portion of chromatograms) for the holo samples but not for the apo samples. DNase activity emerges concomitantly with dimers, as evidence by the disappearance of the full-length DNA oligonucleotide and appearance of shorter nucleotide products (black portion of chromatograms). Uncleaved oligonucleotide and the closed monomer I form of the protein are marked for reference.

We developed an SEC assay for simultaneously monitoring protein conformation and DNase activity of the SF series. SF70-NFM is mixed with SF70-CFM (5 μM each) and FK506 is added at time zero. The enzymes are allowed to swap for the indicated times, at which point a 23-mer single-stranded DNA oligonucleotide is added, the enzymatic digestion is allowed to proceed for 5 min, and the reaction is quenched by EDTA. The mixture is then injected onto an SEC column. All forms of the protein (colored red in Fig. 4) elute earlier than the uncleaved oligonucleotide and its fragments (colored black). As swapping proceeds, new protein and DNA species become further separated from each other, as protein oligomers elute earlier than the parent monomer and digested DNA products elute later than the parent oligonucleotide.

In the absence of FK506, the mixture of SF70-NFM and SF70-CFM remains mostly in the inactive closed monomer I form (MW_app_ = 32.0 kDa) with only minor populations of higher order species present (Fig. 4). A small amount of active, swapped species is detected 2 h after mixing, as indicated by the reduction of the full-length oligonucleotide peak and appearance of smaller DNA products. By contrast, when FK506 is added, the full-length oligonucleotide peak disappears within 20 min of swapping, and mostly short nucleotide products are detected after 1 h. The closed monomer I peak shifts slightly but significantly to the right (MW_app_ = 29.3 kDa) within 5 min of FK506 addition. We attribute this new peak to closed monomer II, which might be expected to elute as a more compact species due to folding of the FKBP domain. A larger species (MW_app_ = 51 kDa) also appears at this time point, but since DNase activity is not detected it likely consists of open monomers that elute close to the same size as swapped dimers by virtue of the SNase domain being at least partially unfolded as suggested by the CD data (Fig. 2A). Figure 4 demonstrates that FK506 substantially increases the population of the catalytically active species of SF70, on a time scale consistent with that observed in the CD kinetic experiments of Fig. 2A.

The SEC assay reveals similar trends for SF53. The mixture of SF53-NFM and SF53-CFM remains mostly as closed monomer I (MW_app_ = 32.3 kDa) without FK506 (Fig. S2). Upon adding FK506, we more clearly observe the rapid conversion of closed monomer I to closed monomer II (MW_app_ = 24.1 kDa). In agreement with the AUC data, SF53 swaps more slowly and/or to a lesser extent than does SF70. After 4 h of swapping only ~1/3 of SF53 molecules elute as dimers/open monomers (MW_app_ = 46.6 kDa), and less of the DNA substrate is degraded compared to SF70. At this time point higher-order oligomers appear in the chromatogram as a broad tail leading up to the dimer peak, again consistent with the AUC results.

### Fluorescence assay for DNase activation

To quantitatively measure the kinetics of FK506-induced swapping, and to determine the resulting fold-change of enzymatic activity, we employed a fluorescence-based DNase assay. A mixture of >40 random, single-stranded DNA oligonucleotides of 25–35 bases in length is mixed with Sytox green, a dye that exhibits a 500-fold increase in fluorescence when intercalated into DNA^29^, at an average ratio of ~0.6 molecules of Sytox per oligonucleotide. NFM and CFM variants are mixed 1:1 at 4 μM, 10 μM, and 20 μM total enzyme concentration and FK506 is added at time zero. At the indicated times an aliquot is added to the DNA/Sytox mixture and degradation is monitored by loss of fluorescence. Controls with WT SNase show that the initial velocity of the reaction is proportional to enzyme concentration (Fig. S3), making the assay suitable for determining the extent of domain swapping. One notable difference between the fluorescence and SEC assays is that they report on different stages of the enzymatic reaction. Fluorescence detects only those cleavage events that result in Sytox release, i.e. degradation to short or possibly single nucleotides. SEC reports on earlier events—mainly initial cleavage of the full-length substrate into relatively large products—with subsequent reactions producing species too small to be resolved on the column.

**Figure 5:**
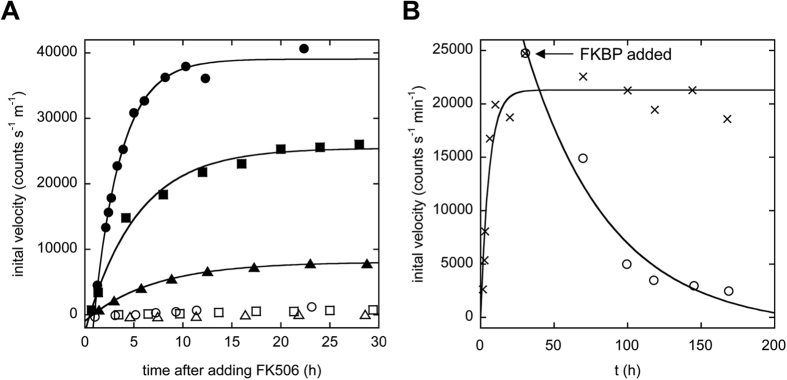
FK506 increases DNase activity of SF70 by ~100-fold and in a reversible manner. (**A**) Closed and open symbols indicate that FK506 or DMSO vehicle, respectively, was added to a 1:1 mixture of SF70-NFM and SF70-CFM at time zero. Circles, squares, and triangles designate total enzyme concentrations of 20 μM, 10 μM, and 4 μM, respectively. (**B**) Reversibility is demonstrated by adding FK506 to a mixture of SF70-NFM and SF70-CFM (10 μM) as in panel A, waiting until full enzymatic activity is reached (arrow), and then adding a 10-fold excess of free FKBP to compete off FK506.

FK506 induces DNase function robustly at all three concentrations of SF70 (Fig. 5A). By contrast, enzymatic activity is barely detectable in the corresponding apo SF70 controls. The change in activity can be estimated by dividing the initial velocity of the holo enzyme by that of the apo enzyme, and averaging this value for the last three time points of each curve. This calculation yields fold-increases of 136 ± 13, 41 ± 8.4, and 57 ± 25 for the 4 μM, 10 μM, and 20 μM enzyme samples, respectively. The large uncertainties in these figures are due to the activities of the apo enzymes being at near-background levels.

The Sytox assay finds that SF53 remains essentially inactive after addition of FK506 (Fig. S4). This result is unexpected because AUC and SEC experiments indicate that FK506 induces SF53 to oligomerize and partially degrade the full-length oligonucleotide (albeit to lesser extents than SF70). The lack of fluorescence-monitored activity may reflect the inability of swapped SF53 to degrade DNA to short or single nucleotides. Position 53 in SNase is adjacent to the substrate binding groove whereas position 70 is distant (Fig. 1A) and this might explain why insertion of FKBP at position 53 could potentially alter the specific activity of SNase.

We previously showed that lever-target pairs in which the lever is inserted into two different positions of the target can domain swap with each other^17^. Moreover, these hetero-swapped complexes can form with greater propensity than their respective homo-swapped counterparts. For example, SF53 by itself can only swap if the polypeptide strands cross over at position 53, and SF70 in isolation can only swap by crossing over at position 70. When SF53 and SF70 are mixed, they have the additional option of forming a heteroswapped complex in which the hinge can be anywhere between positions 53 and 70 (Fig. S5A). The mixture of 5 μM SF53-NFM and 5 μM SF70-CFM exhibits a (28 ± 4.8)-fold increase in DNase activity on FK506 addition (Fig. S6A). This value indicates that a significant fraction of molecules hetero-swap, although the exact percentage cannot be determined since the crossover point and specific activity of the resulting hetero-swapped complex are unknown. As a negative control we tested the ‘reverse’ mixture of SF70-NFM and SF53-CFM. The hetero-swapped dimer in this case contains both NFM and CFM mutations (Fig. S5B). As expected, no activity is observed with or without FK506 even after 50 h of incubation (Fig. S6B).

### Regulation of RBP ribose binding activity by FK506

**Figure 6:**
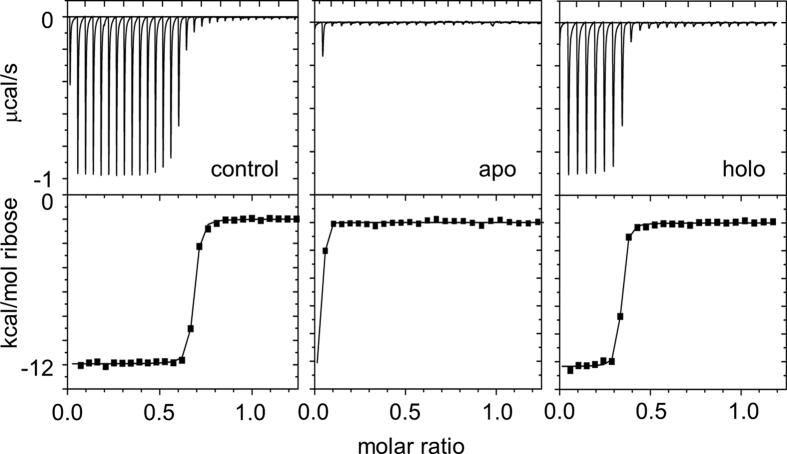
FK506 increases ribose binding activity of RF34-NFM + RF125-CFM by >15-fold. ITC thermograms (top) and fitted ribose binding curves (bottom) are shown for RF125 (left), apo RF34-NFM + RF125-CFM (middle), and holo RF34-NFM + RF125-CFM (right). Fitted K_d_ and binding stoichiometry values are indicated in the text.

To address the question of whether FKBP has general utility as a triggerable lever, we inserted it into RBP and tested if FK506 can regulate ribose binding (Fig. 1A). We chose positions 34 and 125 for insertion because in our earlier study using ubiquitin as the lever we found that those sites result in efficient hetero-swapping with the hinge region being at position 95^17^. To quantify ribose binding we titrated a mixture of RF34-NFM + RF125-CFM (20 μM each) with ribose and measured the change in enthalpy by isothermal titration calorimetry (ITC). The positive control, RF125 without the NFM and CFM mutations, binds with a fitted stoichiometry (*n*) of 0.74 ± 0.08 mole ribose per mole of protein monomer with a dissociation constant (K_d_) of 12.8 ± 0.13 nM (Fig. 6). In the absence of FK506, only a minor fraction of the RF34-NFM + RF125-CFM mixture is competent to bind ribose. The first ribose injection releases a small amount of heat; no further enthalpy change is observed for subsequent injections indicating saturation. K_d_ and *n* cannot be determined accurately from these data, but fixing K_d_ to 17.2 nM (the same value obtained from the holo sample below) yields an upper estimate of *n* ≤ 0.02. When FK506 is added, a full binding curve is restored with *n* = 0.32 ± 0.05 and K_d_ = 17.2 ± 7.6 nM as the fitted parameters. Recalling that at most 50% of the positive control activity can be regained by swapping (Fig. 1C), and that homo-swapped complexes are non-functional, the observed stoichiometry indicates that FK506 triggered nearly all monomers to hetero-swap. This amounts to a ≥15-fold activation of ribose binding activity.

### Reversibility of switching

In order to demonstrate reversibility of the SF70 switch, we used a 10-fold excess of free FKBP to compete off FK506 that was bound to the swapped complex of SF70-NFM/SF70-CFM, and measured loss of enzymatic activity using the Sytox assay (Fig. 5B). DNase activity is reduced by 50% after ~40 h and returns to baseline levels by ~100 h, demonstrating full albeit slow reversibility. The control sample in which no FKBP was added retains full activity during this time. As an additional control, we asked whether FKBP could be causing SF70 to lose function by an artifactual means (e.g. inducing SF70 to unfold, aggregate, or become degraded) rather than by shifting the population of SF70 back to monomers. We treated WT SF70 with FK506 then FKBP as described above, then tested for DNase activity. This enzyme hydrolyzed DNA at the same rate as the control sample in which no FK506 or FKBP was added (Fig. S7), indicating that FKBP does not cause SF70 to misfold or otherwise become inhibited.

We tested for reversibility of the RF switch using a similar approach but with SEC as the readout. RF34-NFM, RF125-CFM, and FK506 were mixed and incubated at 37 °C. After 24 h, the majority of molecules elute as tetramers, with a minor population of dimer and a trace amount of monomer (Fig. S8). After adding excess FKBP to abstract FK506 from the complexes, the tetramer peak reverts to an approximately equal mixture of dimer and monomer by 25 h, and to mostly monomer by 43 h.

The reverse rates are considerably slower than the forward rates for both SF and RF switches. Unlike binding of FK506, which introduces conformational strain into the monomeric proteins, removal of FK506 does not generate strain in the swapped complexes. De-swapping is thus governed by simple dissociation to the closed monomer I form. This process is expected to be slow because it requires that both of the swapped SNase or RBP domains within each dimer unfold approximately simultaneously.

## Discussion

The combination of CD, AUC, SEC, and functional assays provide definitive evidence for INDOS (Fig. 1). To our knowledge this is the first example of rational design in which a small molecule is used to trigger protein domain swapping and subsequent activation of function. Although INDOS is new as a protein engineering tool, it evokes an important class of biological switches: receptor tyrosine kinases (RTKs). RTKs mediate signal transduction by dimerizing on the plasma membrane in response to binding their cognate ligands. RTK monomers are inactive as kinases; only by dimerizing do the monomers cross-phosphorylate each other and become functional enzymes. RTKs do not domain swap—they associate by conventional means—but RTKs and INDOS both use ligand binding to drive conformational changes that perturb an existing monomer/dimer equilibrium toward the latter.

INDOS is distinct from RTKs in that the mechanism that links binding to dimerization is simpler, easier to design, and modular. The two main requirements for the recognition module are that it must possess a relatively long N-to-C terminal distance (≥25 Å) and be significantly stabilized by ligand binding. Our results establish FKBP as one such module that can potentially be used to construct *in vivo* switches that respond to the cell-permeant drugs FK506 and rapamycin. One can conceive of other levers that respond to different effectors (small molecule or protein ligands, changes in pH or temperature, or light), as long as they are stabilized by their respective effector signals and satisfy the N-to-C terminal distance criterion.

The versatility of INDOS, however, lies in the diversity of biological functions that can potentially be regulated by plugging the recognition module into a variety of target proteins. The requirements for the target are that it: (i) domain swap in response to ‘pulling’ stress applied by the lever, and (ii) that its function can be knocked out by either one of two mutations, one placed N-terminal to the lever insertion point and the other placed C-terminal to the lever insertion point. With regard to the former, the results of this study and others suggest that domain swapping is a common route that different proteins take to resolve lever-induced conformational stress. Even if one lever placement fails, proteins typically contain many potential insertion sites (i.e. surface loops/turns) at least one of which will result in efficient swapping^17^,^22^. For example, as part of this study we also inserted FKBP into position 78 of SNase to create SF78. SEC experiments revealed that SF78 remained monomeric with and without FK506 (not shown). When constructs fail to swap the reason is often unclear, but in this case it is likely that the loop surrounding residue 78, being the largest in SNase, is long enough to decouple the pulling force exerted by the FKBP domain^21^. SF53 exhibits the opposite behavior: it tends to domain swap even in the absence of FK506 and it forms higher-order oligomers compared to SF70 (Fig. S1). That swapping is enhanced by placing the lever in certain sites over others appears to be a common result. Fusing the ubiquitin lever in RBP^17^ and barnase^30^ results in constructs that form mainly swapped dimers or a mixture of higher-order oligomers, depending on the insertion loop. The basis for this behavior is not obvious from inspection of structural features such as loop length or proximity to either terminus of the protein. We previously speculated that the extent to which lever-target constructs oligomerize may be dictated by steric crowding at the hinge region, i.e., how well the lever domains pack with each other and with the target domains in the swapped complexes^17^.

Once a lever-target pair has been chosen, their relative thermodynamic stabilities must be balanced such that the lever domain in its apo state is less stable than the target domain. This condition ensures that the fusion protein will adopt the closed monomer I conformation in the absence of ligand. For this reason we needed to introduce the V24A mutation into the FKBP domains of all constructs. The V24A mutation destabilizes FKBP by 3.2 kcal/mol without affecting its affinity for FK506^31^. After the lever domain binds the ligand, it is desirable but not essential that it be more stable than the target domain. All that is required is that the lever gain enough stability such that it destabilizes the target in its monomeric form to the point where swapping is favored, according to the antagonistic coupling model that we presented previously^21^. Using the switches developed here as guideposts for future designs, the folding free energy (ΔG_fold_°) of free FKBP (V24A mutant) is 2.9 kcal/mol and this value increases by 2.7 kcal/mol in the presence of ligand^31^,^32^. The target protein is always destabilized by insertion of the lever, and wild-type SNase (ΔG_fold_° = 5.5 kcal/mol) proved to be too unstable to fold. We therefore employed the ΔPHS variant of SNase (ΔG_fold_° = 12 kcal/mol^33^). The NFM and CFM mutations decrease ΔG_fold_° by 1.4 kcal/mol and 0.9 kcal/mol, respectively^28^. Wild-type RBP has the opposite problem: it is so stable, and unfolds so slowly, that ΔG_fold_° cannot be measured accurately by chemical denaturation. Our initial RF constructs failed to swap in response to FK506, most probably because the RBP domain was too stable and/or unfolded too slowly. Fortunately, it is usually straightforward to tune a protein’s stability by altering amino acids in its hydrophobic core, and introducing the destabilizing I236A mutation into RF34-NFM and A29T + V52E mutations into RF125-CFM resolved the unfolding kinetic trap.

INDOS causes proteins to self-assemble and this feature may be useful for generating active biomaterials that form in response to a small molecule or other trigger. With regard to the functional switching capability presented here, one limitation of INDOS is that activation requires dimerization (or higher) and protein concentration must therefore be above the apparent K_d_ of dimerization. SF and RF switches are effective at low micromolar concentration; we have not yet established the lower limit of protein concentration. It should be possible, however, to combine INDOS with the alternate frame folding (AFF) design that we developed earlier^27^,^34^,^35^ to create a unimolecular switch that operates at all protein concentrations. Using SF70 as an example, FKBP is inserted into position 70 of SNase as in Fig. 1. The segment of SNase from residue 71 to its C-terminus is then duplicated and appended to the N-terminus of the FKBP-SNase fusion protein via a peptide linker long enough to bridge the N- and C-termini of SNase. The resulting protein can interconvert between two folds: one in which the apo FKBP domain is unfolded and the SNase domain adopts its normal fold (closed monomer I with the duplicated SNase segment extending from the N-terminus), and the other in which FK506-induced folding of the FKBP domain forces the SNase domain to adopt a circularly permuted structure with the duplicated segment extending from the C-terminus. The normal and circularly permuted forms are mutually exclusive, monomeric, and unable to swap. We are currently testing this hybrid INDOS-AFF design.

## Experimental Procedures

### Protein expression and purification

Human FKBP12 DNA sequences (containing the V24A and C22A mutations) were inserted into the specified positions of the staphylococcal nuclease gene (ΔPHS variant) and the *T. tengcongensis* RBP gene according to the procedure of Geiser *et al*.^36^. All genes were fully sequenced. Proteins were expressed in *E. coli* BL21(DE3) with induction of isopropyl β-D-thiogalactopyranoside occurring at 20 °C for 12–15 hr. Cell pellets were resuspended in 10 mM Tris (pH 7.5), 0.3 M NaCl, and lysed using a small amount of lysozyme followed by sonication. The soluble fraction of the lysate was loaded onto a nickel-nitrilotriacetic acid column (Bio-Rad) and proteins were purified following the manufacturer’s protocols. For RF variants that did not contain NFM or CFM mutations, we incorporated an additional wash with 10 mM Tris (pH 7.5) and 6 M guanidine hydrochloride to denature the proteins and remove bound ribose. Eluted proteins were dialyzed against double-distilled H_2_O and lyophilized. FKBP was purified in the same manner except the soluble fraction of the lysate was passed through a Q-Sepharose column to remove excess DNA, acetic acid was added to adjust pH to 5.0, and the solution was centrifuged to remove the precipitated material. The supernatant was loaded onto a SP-Sepharose column, followed by washing with 20 mM sodium acetate (pH 5.0), and elution with a linear gradient of 0 to 1 M NaCl. FKBP fractions were pooled, dialyzed against double-distilled H_2_O, and lyophilized. All proteins were judged to be >95% pure by SDS- PAGE with coomassie staining.

### Circular dichroism

Protein samples were prepared in 10 mM Tris (pH 8.0), 0.15 M NaCl, and 10 mM CaCl_2_. FK506 (LC Laboratories) was added by a 2000-fold dilution from a 100 mM stock solution in DMSO. CD data were recorded on an Aviv model 420 instrument. Experimental temperature was 25 °C.

### Sedimentation velocity analytical ultracentrifugation

FK506-containing samples were prepared by adding 50 μM FK506 to SF70 and SF53 and incubating for 24–48 h at room temperature prior to AUC experiments. Samples were loaded into cells containing 12 mm two-sector charcoal-filled Epon centerpieces and quartz windows and inserted into a four-hole An-60 Ti rotor and run in a Beckman-Coulter ProteomeLab XL-A analytical ultracentrifuge equipped with absorbance optics. Samples were scanned 300 times with a 0 time interval between scans (total run time ~15.5 h). Raw data were analyzed by the continuous c(s) distribution method in the program SEDFIT^37^. For each sample, 80–100 scans were loaded (omitting every other scan) and the data were fit using alternating rounds of Simplex and Marquardt-Levenberg algorithms, achieving root-mean squared deviation values of ≤0.01. Experimental sedimentation coefficients were converted to standard conditions at 20 °C in water (S_20,w_) using the program SEDNTERP^38^. Frictional ratios (f/f0) were also calculated via SEDNTERP, using both the expected molecular weight and partial specific volume of the proteins based on the primary amino acid sequence. Experimental temperature was 25 °C.

### Size exclusion chromatography

NFM and CFM variants (5 μM each) were mixed together in SNase reaction buffer (25 mM Tris pH 8.5, 0.15 M NaCl, 10 mM CaCl_2_) and incubated overnight at room temperature. FK506 (50 μM) was added at time zero. At the indicated time points, an aliquot of this mixture was withdrawn and 5 μM substrate (the single-stranded DNA oligonucleotide 5′-GCCCTCTTTCATTGCACCAGAGC-3′) was added. The DNase reaction was allowed to proceed for 5 min at room temperature then terminated by addition of EDTA. Samples were then injected onto a Superdex S200 Increase SEC column (GE Healthcare) employing the same buffer as above except containing 1 mM EDTA instead of 10 mM CaCl_2_.

### Fluorescence assay for DNase activity

Fluorescently-labeled substrate (50× working concentration) was prepared by boiling a mixture of >40 random, single-stranded DNA oligonucleotides of 25–35 bases in length (nucleotide concentration = 7.26 mM) for 1 h in H_2_O, followed by rapid cooling on ice. Sytox green was then added to a final concentration of 150 μM, or ~0.6 mol Sytox per mol oligonucleotide. Enzymes were mixed in the same reaction buffer as in SEC experiments and divided into two identical tubes. At time zero, FK506 (50 μM) was added to one tube of enzyme and an identical volume of the DMSO vehicle was added to the other. At the indicated time points, an aliquot of enzyme was removed, 1/50 volume of 50× substrate was added, and the DNase reaction was monitored by loss of Sytox fluorescence at 523 nm (with excitation at 504 nm) using a Horiba Fluoromax-4 fluorimeter. Experimental temperature was 20 °C.

Initial velocities were calculated as follows. We observed a minor but unexpected initial increase in fluorescence for some samples prior to the expected decrease. The origin of the increasing fluorescence phase is not known, but its rate increased with increasing enzyme concentration to the point where it disappeared into the mixing dead time for many samples. We therefore calculated initial velocity from the steepest downward slope of the fluorescence signal after the initial increase, if present. Short windows of the decay curve, corresponding to approximately linear segments, were manually selected and fit to a line. The initial velocity is reported as the most negative slope obtained by this procedure.

### Isothermal titration calorimetry

RF34-NFM + RF125-CFM (20 μM each) or RF125 (50 μM) was mixed with 100 μM FK506 or DMSO vehicle and incubated for 24 h at 37 °C. Samples were then dialyzed against 20 mM phosphate (pH 7.0) and centrifuged. Protein concentration was determined from the absorbance of the supernatant (ε_280_ = 14,440 M^−1^ cm^−1^). Data were collected at 25 °C using a MicroCal VP-ITC (Malvern Instruments). Each injection consisted of 5–10 μl of 0.25 mM–0.5 mM ribose, added over 20 s with 240 s between injections. Measured enthalpies were fit to the one-site binding isotherm using the VP Viewer 2000 software package from the manufacturer.

## Additional Information

**How to cite this article**: Karchin, J. M. *et al*. Small Molecule-Induced Domain Swapping as a Mechanism for Controlling Protein Function and Assembly. *Sci. Rep.* **7**, 44388; doi: 10.1038/srep44388 (2017).

**Publisher's note:** Springer Nature remains neutral with regard to jurisdictional claims in published maps and institutional affiliations.

## Supplementary Material

Supplementary Figures and Table
