# Supplementary material for: Small Molecule-Induced Domain Swapping as a Mechanism for Controlling Protein Function and Assembly
Source: Sci Rep. 2017 Mar 13;7:44388. doi: 10.1038/srep44388 (PMC5347425; doi:10.1038/srep44388)
Supplement: Supplementary Figures and Table [file srep44388-s1.pdf]

Supplementary Information

"Small Molecule-Induced Domain Swapping as a Mechanism for Controlling Protein Function and Assembly"

Joshua M. Karchin, Jeung-Hoi Ha, Kevin E. Namitz, Michael S. Cosgrove, and Stewart N. Loh

Contents:

Figure S1. Oligomerization of SF53 in the presence and absence of FK506 analyzed by AUC

Figure S2. FK506-induced oligomerization and activation of SF53 monitored by SEC

Figure S3. Standard curves for the Sytox activity assay showing that initial velocity is proportional to SNase concentration

Figure S4. Sytox DNase activity assay of SF53

Figure S5. Schematic of forward and reverse heteroswaps, using SF53 and SF70 as an example

Figure S6. FK506 causes the mixture of SF53 and SF70 to heteroswap and gain function

Figure S7. Reversibility of SF70: FKBP does not cause WT SF70 to lose activity

Figure S8. Reversibility of the RF34 + RF125 ribose binding switch demonstrated by SEC

Table S1. Fitted parameters from SEC experiments

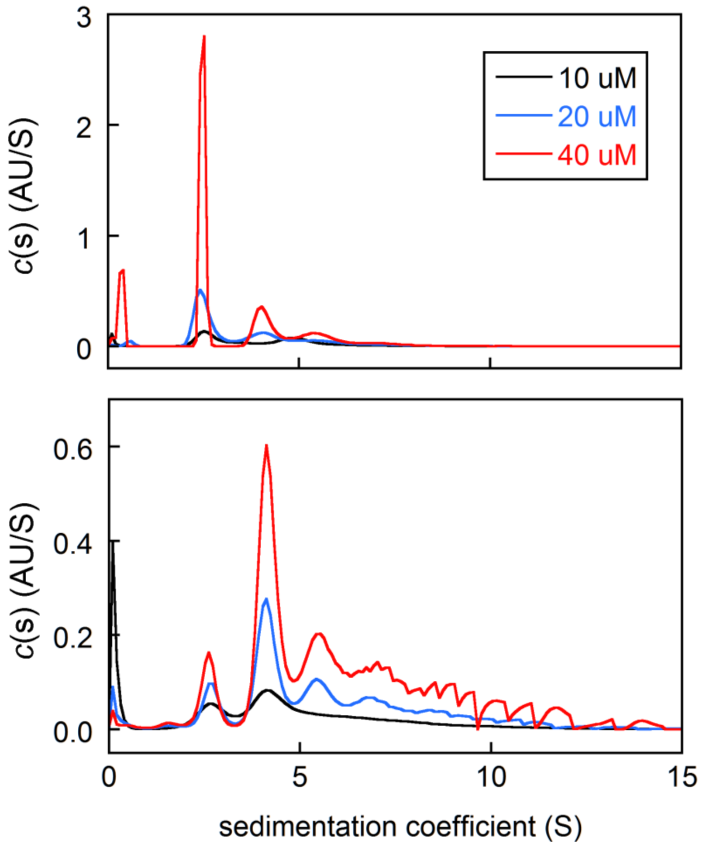

Figure S1. Oligomerization of SF53 in the presence and absence of FK506 analyzed by AUC. Sample and experimental conditions are identical to those in Fig. 2B. The apo and holo samples are on top and bottom, respectively.

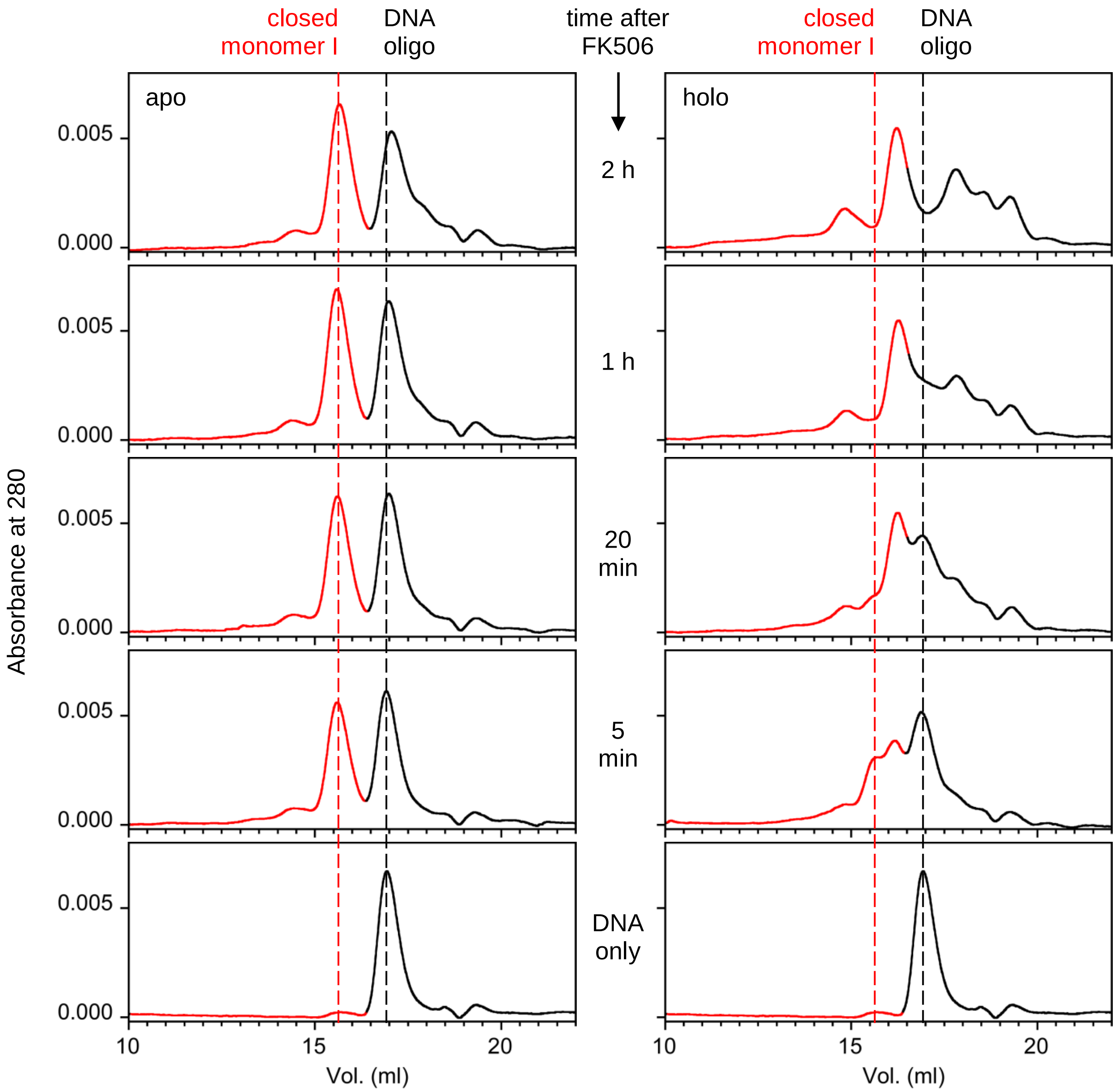

Figure S2. FK506-induced oligomerization and activation of SF53 monitored by SEC. Following addition of FK506 or DMSO vehicle to a mixture of SF53-NFM + SF53-CFM, size exclusion chromatograms show conversion of protein monomers to domain-swapped dimers and oligomers (red portion of chromatograms), and degradation of full-length DNA oligonucleotide to shorter nucleotide products (black portion of chromatograms). Uncleaved oligonucleotide and the closed monomer I form of the protein are marked for reference.

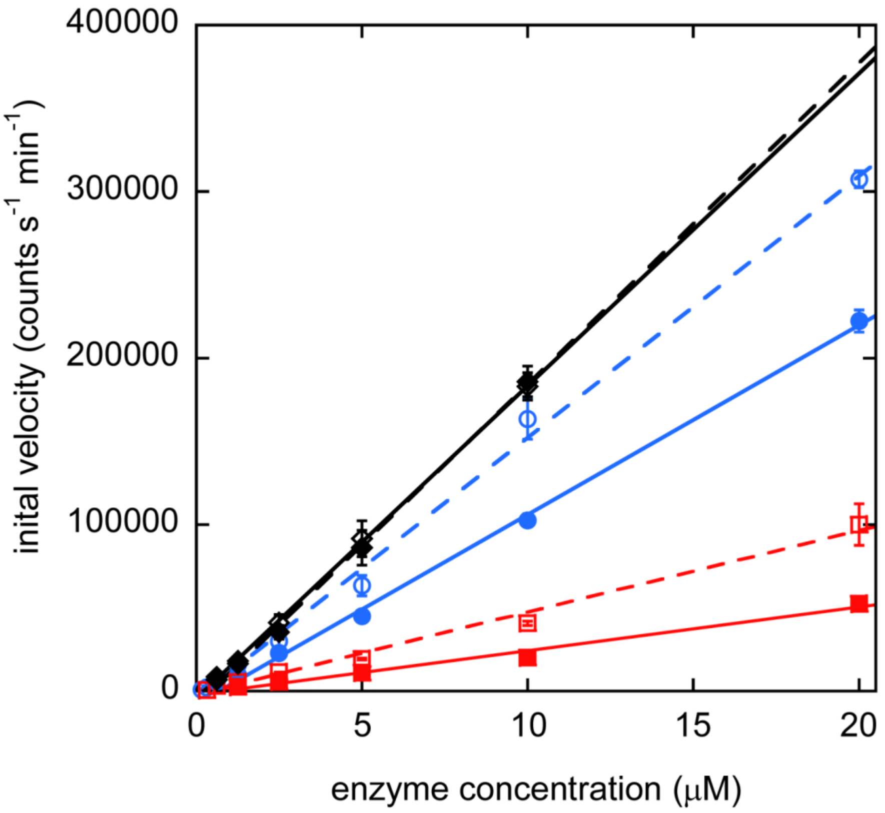

Figure S3. Standard curves for the Sytox activity assay showing that initial velocity is proportional to SNase concentration. SNase ΔPHS (black), SF70 (blue), and SF53 (red) were mixed with Sytox green and initial velocities were recorded as described in *Experimental Procedures*. Open symbols/dashed lines and closed symbols/solid lines indicate the absence and presence of FK506 (50 μM), respectively. Lines are linear fits and error bars are the standard errors of at least three measurements.

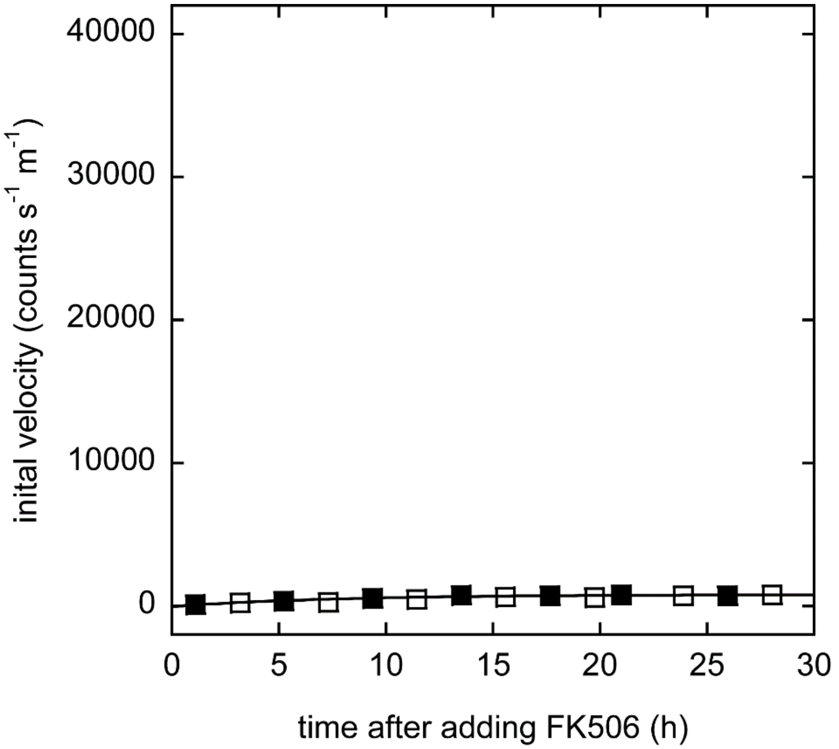

Figure S4. Sytox DNase activity assay of SF53. Closed and open squares indicate that FK506 or DMSO vehicle, respectively, was added to a mixture of SF53-NFM + SF53-CFM (5 μM each) at time zero. The Y-axis is scaled to match that in Fig. 4A.

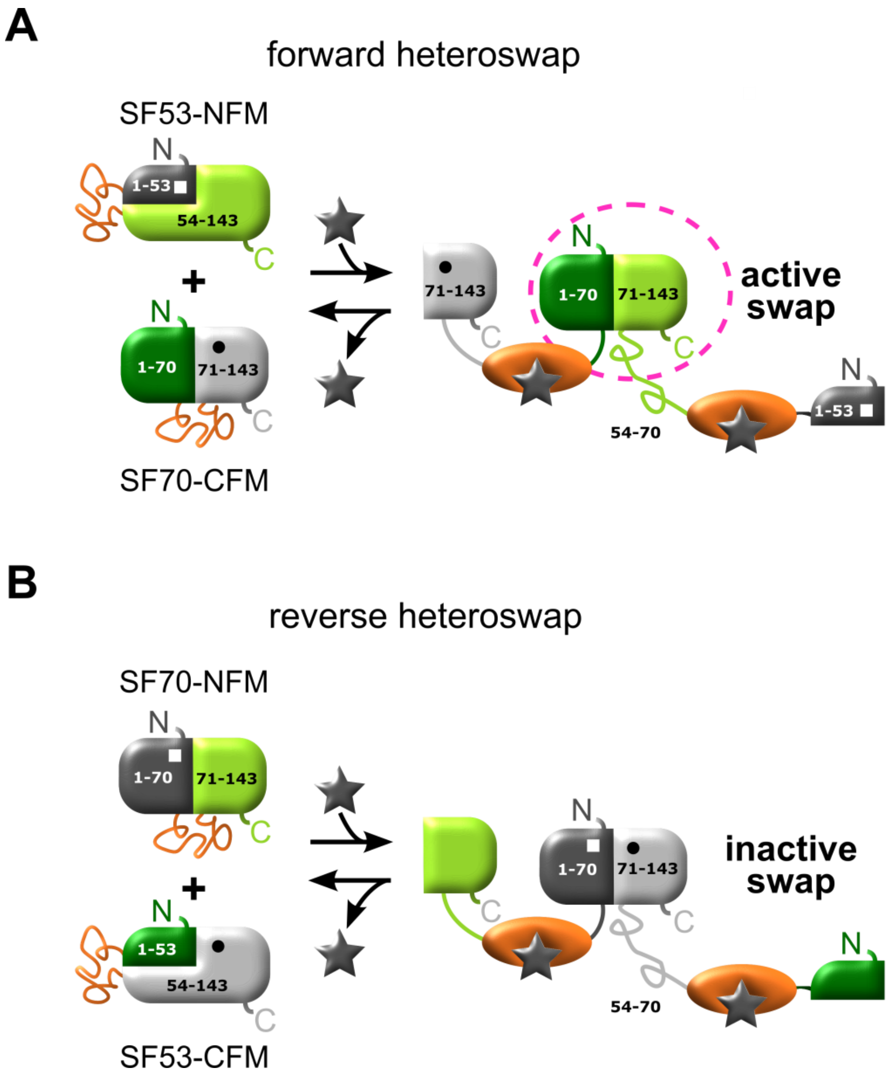

Figure S5. Schematic of forward and reverse heteroswaps, using SF53 and SF70 as an example. The heteroswap is shown with the crossover occurring at position 70, but the location of the hinge can be anywhere between positions 53 and 70. The swapped, enzymatically active SNase domain is depicted as the fully green structure.

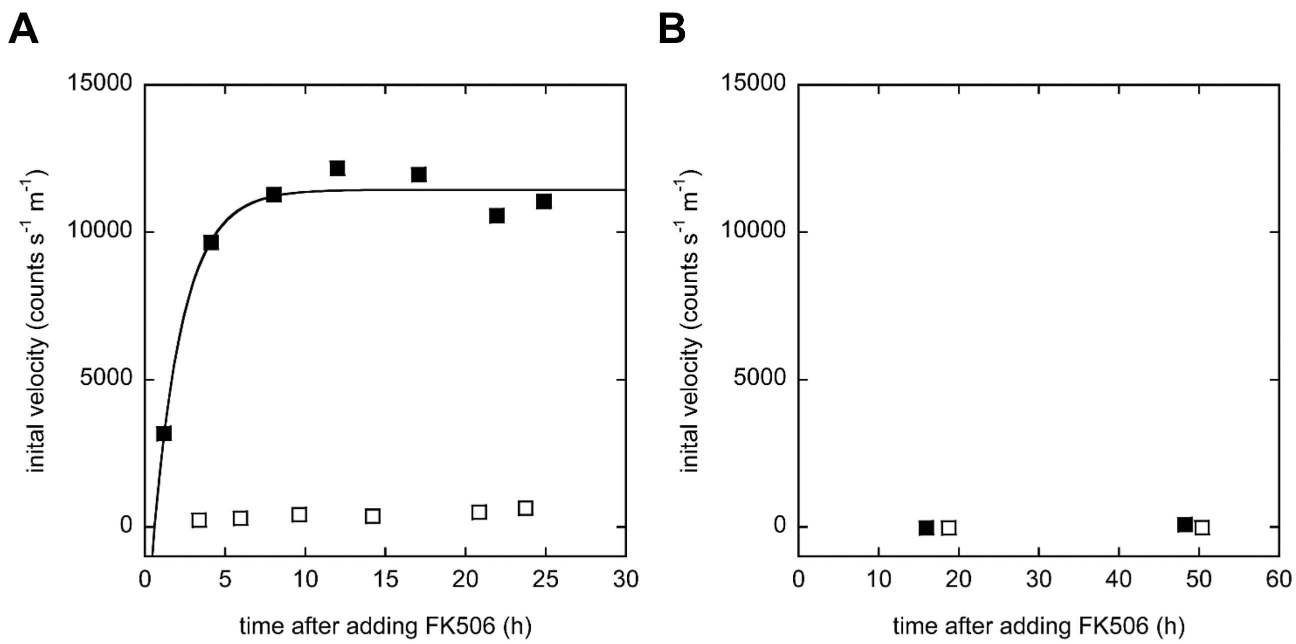

Figure S6. FK506 causes the mixture of SF53 and SF70 to heteroswap and gain function. **(A)** Heteroswapping is revealed by FK506 triggering of DNase activity when the functional mutations are placed in the forward orientation (SF53-NFM + SF70-CFM) **(A)**, but not when they are placed in the reverse orientation (SF53-CFM + SF70-NFM) **(B)**. Open and closed squares indicate the absence and presence of FK506 (50 μM), respectively. Enzyme concentrations are 5 μM SF53 and 5 μM SF70.

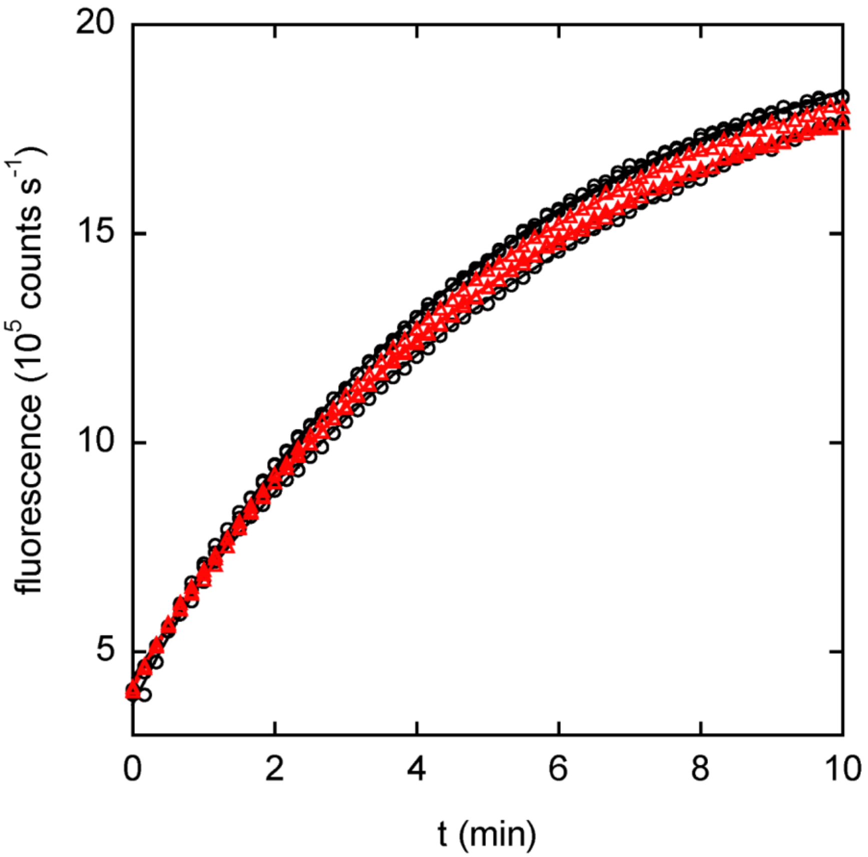

Figure S7. Reversibility of SF70: FKBP does not cause WT SF70 to lose activity. WT SF70 (2 μM) was mixed with FK506 (3 μM) and incubated at room temperature for 15 h in 10 mM Tris (pH 7.5), 10 mM $CaCl_2$. FKBP (20 μM) was then added and allowed to compete for FK506 binding for 48 h. To quantify DNase activity, 0.1 μM enzyme was added to 1.9 μM of the ssDNA substrate 5'-(6-carboxy fluorescein)-CATCTTAAACTG-(5-carboxy tetramethylrhodamine)-3'. Cleavage of the substrate was monitored by increase in fluorescence of the 6-carboxy fluorescein moiety (emission at 517 nm, with excitation at 487 nm), as it became de-quenched by the 5-carboxy tetramethylrhodamine group (red triangles). The control sample (black circles) consists of the same enzyme to which no FK506 or FKBP was added. Triplicate measurements of each sample are shown. Solid lines are best fits of the data to a single exponential function. The fitted rate constants are 0.177 ± 0.035 min^{-1} (red triangles) and 0.179 ± 0.027 min^{-1} (black circles).

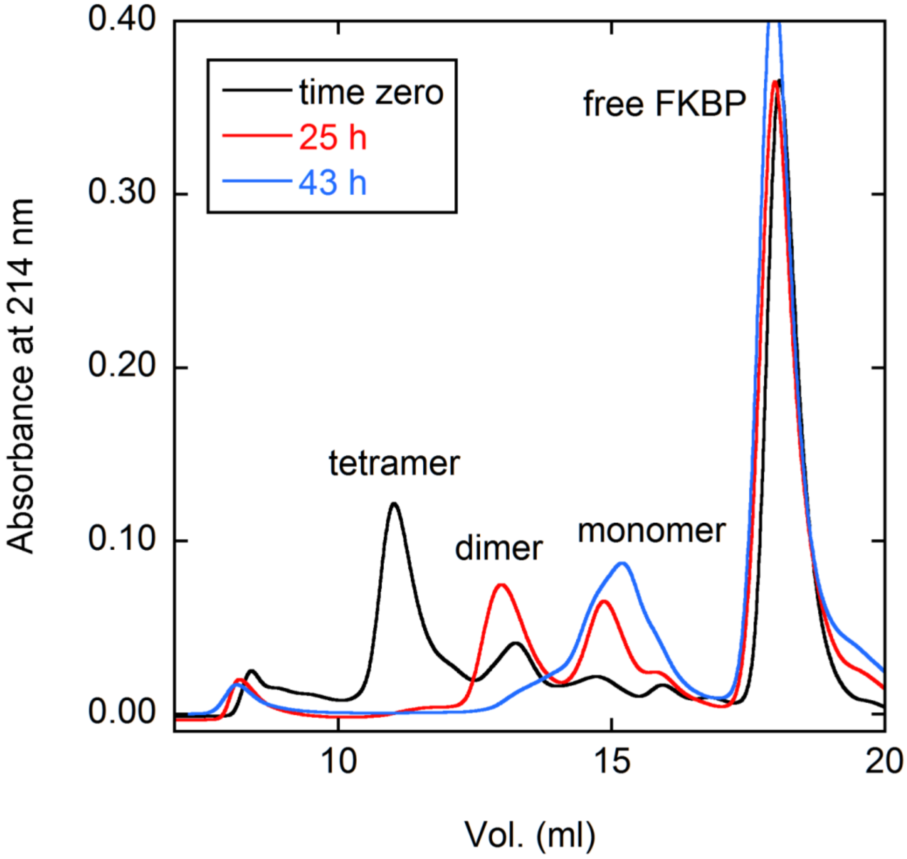

Figure S8. Reversibility of the RF34 + RF125 ribose binding switch demonstrated by SEC. Holo complexes (black) were generated by adding FK506 (50 μM) to a mixture of RF34-NFM + RF125-CFM (20 μM each). Addition of 100 μM FKBP causes the tetramers to dissociate into dimers and monomers after 25 h (red), and to mostly monomers after 43 h (blue).

**Table S1. Fitted parameters from SEC experiments.**				
Protein variant	Protein conc. (μM)	Peak 1 Sed. coeff. (S), MW_{app} (kDa), % total area	Peak 2 Sed. coeff. (S), MW_{app} (kDa), % total area	Peak 3 Sed. coeff. (S), MW_{app} (kDa), % total area
SF70 apo	10	3.09, 32.7, 90.1	NA	NA
SF70 apo	20	2.56, 25.7, 77.5	4.20, 54.1, 22.0	NA
SF70 apo	40	2.60, 25.0, 75.6	4.27, 52.8, 24.4	NA
SF70 holo	10	2.43, 25.5, 6.13	4.07, 55.3, 78.7	NA
SF70 holo	20	2.41, 23.7, 4.55	4.05, 51.6, 81.0	NA
SF70 holo	40	2.49, 25.4, 3.28	4.06, 52.9, 79.9	NA
SF53 apo	10	2.81, 42.0, 48.3	5.24, 107, 46.1	NA
SF53 apo	20	2.53, 39.2, 54.2	4.14, 81.8, 26.4	NA
SF53 apo	40	2.47, 25.2, 51.0	4.12, 54.1, 19.5	NA
SF53 holo	10	2.67, 39.6, 17.2	5.76, 125, 62.6	NA
SF53 holo	20	2.69, 34.4, 10.2	4.17, 66.4, 33.4	5.55, 101, 20.5
SF53 holo	40	2.60, 31.3, 7.1	4.19, 64.0, 32.7	5.62, 99.2, 19.2
